# Antibiotic resistance of urinary pathogens after kidney transplantation: a 10-year single-center survey in Germany

**DOI:** 10.1007/s15010-025-02493-0

**Published:** 2025-03-10

**Authors:** P. Weber, P. Braß, J. Jäger, L. Jacquet, S. Jansen, A. Gäckler, C. Jürgens, J. Reinold, U. Eisenberger, P.-M. Rath, A. Kribben, O. Witzke, H. Rohn

**Affiliations:** 1 Department of Infectious Diseases, West German Centre of Infectious Diseases, University Hospital Essen, University Duisburg-Essen, Hufelandstrasse 55, 45147 Essen, Germany; 2 Department of Nephrology, University Hospital Essen, University Duisburg-Essen, Essen, Germany; 3 Department of Nephrology and Rheumatology, Knappschaftskrankenhaus Bottrop, Bottrop, Germany; 4 Institute of Medical Microbiology, University Hospital Essen, University Duisburg-Essen, Essen, Germany

**Keywords:** Urinary tract infections, Kidney transplantation, Antibiotic resistance, Antimicrobial stewardship

## Abstract

**Purpose:**

Urinary tract infections (UTIs) are common complications after kidney transplantation (KT), often resulting in severe outcomes like acute graft failure and sepsis. Factors such as diabetes, age, sex, and type of transplantation significantly influence disease progression. Rising antibiotic resistance complicates treatment, emphasizing the importance of Antimicrobial Stewardship (AMS), particularly during the post-transplant immunosuppression phase. Recent changes in treatment protocols, including a shift away from treating asymptomatic bacteriuria and modifications in antibiotic prescribing, highlight the need for updated resistance trend analyses.

**Methods:**

This retrospective study at the University Hospital Essen analyzed urine samples from kidney transplant outpatients from 2013 to 2022. Pathogen identification and resistance testing focused on common UTI pathogens, including *Escherichia coli*, *Klebsiella spp*., *Pseudomonas aeruginosa*, *Enterococcus faecium*, and *Enterococcus faecalis*. Data on antibiotic prescriptions were sourced from the North Rhine Association of Statutory Health Insurance since 2017.

**Results:**

Out of 10,508 urine samples collected from 6962 patients, bacterial growth was detected in 4126 samples (39%). *Escherichia (E.) coli* was the most frequent pathogen (41%). *Klebsiella spp*., which accounted for 11.7% of all pathogens, showed increasing resistance to piperacillin/tazobactam and ceftazidime. Resistance rates *Enterococcus faecalis* showing a significant decline in levofloxacin (100% resistance in 2014 in all isolates, compared to 2% in 2022). An increasing concern in our cohort is the prevalence of Extended Spectrum Beta-Lactamase (ESBL)-producing Gram-negative pathogens, particularly *Klebsiella spp.,* which are being detected with greater frequency. In our center, we have observed a significant increase in the use of oral antibiotics recommended for first-line therapy. This shift is attributed to updated guidelines and therapeutic recommendations. Consequently, oral cephalosporins are now rarely used due to their low bioavailability.

**Conclusion:**

The study highlights the importance of ongoing surveillance to address antibiotic resistance in KT recipients. Increasing resistance in pathogens like *Klebsiella spp*. necessitates new antimicrobial strategies. Findings should inform future guidelines to preserve antibiotic effectiveness and improve therapeutic outcomes in this vulnerable patient population.

**Supplementary Information:**

The online version contains supplementary material available at 10.1007/s15010-025-02493-0.

## Background

Kidney transplantation (KT) significantly improves the quality of life and survival rates for patients with end-stage renal disease. However, infectious complications, particularly urinary tract infections (UTIs), remain a major challenge, contributing to increased morbidity, mortality, and hospitalization. Among these complications, UTIs are the most common [1, 2].

The incidence and progression of UTIs in KT recipients are influenced by various patient-related factors, including underlying conditions particularly diabetes mellitus, age, type of transplantation, and sex [3]. Bacteriuria can progress to complicated UTIs, potentially compromising allograft function and survival [4, 5]. These infections have a substantial impact on hospitalization rate, graft function outcomes, and mortality in transplant recipients [6, 7]. There is a notable difference in the incidence of UTIs among KT recipients compared to recipients of other solid organs. A cohort study by Vidal et al. (2012), which tracked 2405 solid organ transplant recipients over three years, found that KT recipients had the highest UTI incidence, with 13.84 cases per 100 subjects per year [8]. The early post-transplant period is particularly critical due to intensified immunosuppressive therapy, increasing susceptibility to infections. Prophylactic measures like TMP-SMX are routinely used to prevent Pneumocystis pneumonia, while broad-spectrum antibiotics are often required to manage infections and prevent sepsis.

In 2010, we introduced the Essen algorithm for calculated antibiotic treatment of UTI after KT [9]. This approach addressed both Gram-negative bacteria and the increased incidence of enterococci. It recommended that fluoroquinolones during the initial two months post-transplant, with cephalosporins to be used thereafter. A subsequent re-evaluation of this approach revealed an increased resistance of Gram-negative urinary pathogens, especially *Klebsiella spp*., over a four-year period (2009–2012) in our center leading to a change in in-house practice [10].

Rising antimicrobial resistance complicates UTI management in KT recipients, emphasizing the need for effective prevention and treatment strategies. Understanding local epidemiology and resistance trends is crucial for optimizing therapy. Despite these challenges, there have been several paradigms shifts over the past decade. The question of whether asymptomatic bacteriuria (ASB) should be treated is currently evolving within transplant nephrology. Additionally, prescribing practices have been influenced by new insights into the efficacy and side effects of various antibiotics, including oral cephalosporins and fluoroquinolones. Consequently, the responsible use of antimicrobials, guided by AMS principles, is crucial for mitigating resistance and maintaining the effectiveness of existing treatments [11].

Given these challenges, we analyzed pathogen and resistance trends in KT recipients over a ten-year period (2013–2022) to support the development of targeted treatment strategies.

## Methods

### Study population and design

This single-center study was conducted at the Kidney Transplant Outpatient Department at the University Hospital Essen, Germany. Urine samples from outpatients were retrospectively analyzed to assess the development of microbiological pathogen frequencies and their resistance rates to anti-infective agents. This study included all KT patients who were 18 years or older and attended the outpatient clinic between 2013 and 2022.

Regarding the transplantation, induction therapy for all included transplant patients consisted of steroids and either IL-2 receptor antagonist basiliximab (majority) or anti-thymocyte globulin based on the immunological risk profile. Maintenance immunosuppression included prednisolone, a calcineurin inhibitor and an anti-metabolite. All newly transplanted patients received low-dose TMP-SMX for six months.

In the study, we conducted the analysis on a case-by-case basis, meaning it was not person-specific. A total of 12,692 patient visits were recorded, the median age was 55 years (range, 18–89 years). 5562 cases (44%) were associated with female patients, while 7012 cases (56%) were associated with male patients. In 118 cases (< 1%), no sex was specified.

### Study procedures

The analysis focused on common UTI pathogens: the Gram-negative bacteria *E. coli*, *Klebsiella spp*., *Pseudomonas aeruginosa,* and the Gram-positive bacteria *Enterococcus faecium* and *Enterococcus faecalis.*

The anti-infective therapies evaluated were selected according to local guidelines for the treatment of UTIs and Pneumocystis prophylaxis. Patients with symptomatic UTIs received appropriate antibiotic therapy. Patients with ASB were treated with antibiotics until 2017, after which local guidelines were revised to discontinue treatment for ASB.

The analysis was conducted on a case-by-case basis using all visits to the KT outpatient clinic, which were generated by our hospital’s internal electronic data processing system. Since the evaluation was not patient-specific, patients who presented multiple times may have been included in the analysis multiple times. Additionally, we examined all outpatient cases to determine whether there was a subsequent inpatient admission to our clinic during the observation period. Based on the ICD codes of these inpatient cases, we checked whether a complicated urinary tract infection, sepsis, or bloodstream infection was coded for the stay and evaluated accordingly.

The development of resistances was correlated with antibiotic prescription data exclusively from our KT outpatient clinic. This data was obtained from the North Rhine Association of Statutory Health Insurance (“Kassenärztliche Vereinigung Nordrhein”), which has been legally required to maintain statistics on prescribed antibiotics since 2017. Data for the period from 2013 to 2016 were not available.

This study was approved by the Ethics Committee of the Medical Faculty of the University of Duisburg-Essen (Approval No. 23-11437-BO).

## Treatment

### Local UTI diagnostic and treatment strategy

Until 2017, the strategy followed the so-called “Essen Algorithm,” where a calculated therapy with ciprofloxacin was generously initiated even in cases of ASB or cystitis, especially within the first two months after transplantation [11]. Due to various paradigm and guideline shifts in the treatment of UTIs (for a more detailed explanation, see the discussion), our center has also changed its strategy. Since 2017, ASB is no longer treated, and therapy is administered whenever possible after the pathogen is identified. Similarly, in cases of clinical signs of transplant pyelonephritis or laboratory evidence of infection, therapy is usually initiated based on prior microbiological findings and adjusted upon receipt of current results. Patients with urosepsis are admitted to the hospital and generally receive an empiric therapy with piperacillin/tazobactam, with adjustments based on the antibiogram after the pathogen has been identified.

### Definitions

Symptomatic bacteriuria, ranging from local symptoms, such as cystitis, to the development of pyelonephritis and sepsis, can lead to severe consequences in transplant recipients including impaired allograft function, allograft loss and patient death. According to the recommendations from the Infectious Diseases Community of Practice (IDCOP) of the American Society of Transplantation ASB was defined by the presence of > 10^5^ bacterial colony forming units per millilitre (cfu/ml) in urine without urinary or systemic symptoms of infection. Acute simple cystitis was defined by typical clinical symptoms of lower urinary tract infection without systemic symptoms or inserted foreign material and > 10 white blood cells (WBC)/mm^3^ or > 10^3^ cfu/mL uropathogen. An acute pyelonephritis of the transplant or complicated UTI was defined by fever and other typical symptoms of an acute infection, moreover with pain above the flank or the allocraft or with findings of bacteriemia with same organism as in urine. Laboratory investigations of urine here are defined as > 10 WBC/mm^3^ or > 10^4^ cfu/mL uropathogen. If more than three urogenital tract infections occur within the last 12 months, it is considered a recurrent UTI [12].

Bacteria are further classified according to their resistance mechanisms. Internationally, the term ESBL is commonly used. The classification of multidrug-resistant *P. aeruginosa* (multidrug-resistant (MDR), extensively drug-resistant (XDR), and pandrug-resistant (PDR)) is not entirely consistent. MDR *P. aeruginosa* is defined as non-susceptible to at least one antibiotic in the following classes: penicillins, cephalosporins, fluoroquinolones, aminoglycosides, and carbapenems. XDR refers to susceptibility limited to only two of these antibiotic classes, while PDR describes non-susceptibility to all mentioned antimicrobial agents [13]. In recent years, the term “difficult-to-treat resistance” (DTR) has also been introduced, which refers to a lack of susceptibility to the following agents: piperacillin-tazobactam, ceftazidime, cefepime, aztreonam, meropenem, imipenem-cilastatin, ciprofloxacin, and levofloxacin [14].

### Statistical analysis

Statistical analysis was performed using GraphPad Prism 10.2.3.

Here, the development of resistance between 2013 and 2022 was evaluated. Using the chi-square test, resistant and sensitive pathogens for each antibiotic were compared during the specified period.

The test compared the absolute number of resistant pathogens with the sensitive ones. This approach was also used for the description of the ESBL and MDR isolates. It should be mentioned that the statistics serve as an additional tool to better describe the observed developments. p-values < 0.05 were considered significant.

### Graphic design

The illustrations were generated with GraphPad Prism.

## Results

Between 2013 and 2022, a total of 10,508 urine samples from 6962 patients were analyzed. Bacteriuric episodes, excluding skin and mucosal flora, were observed in 4126 cases (39%) of the samples. While the absolute number of urine samples fluctuated slightly over the observation period, the relative proportion of detected uropathogenic bacteria remained almost constant. An overview is provided in Table 1.

**Table 1:** Number of urine samples and detected bacteriuric episodes 2013–2022: this table shows the absolute numbers of detected uropathogens in all submitted urine samples over time

	Year	Total number of urine samples	Number of bacteriuric episodes	Relative proportion of positive urine cultures
	2013	519	233	43%
	2014	766	287	37%
	2015	1026	387	42%
	2016	1062	442	42%
	2017	1170	466	40%
	2018	1353	530	39%
	2019	1351	583	43%
	2020	1012	380	38%
	2021	1262	482	38%
	2022	987	346	35%
Σ		10,508	4136	39%

The relative proportion of positive urine cultures remained nearly constant throughout the period

### Microbiological profile of UTIs in kidney transplant recipients

Gram-negative bacteria account for more than 75% of cases.

This pattern in the microbiology of UTIs in KT recipient is similar to other patient populations prone to UTIs [15].

### Distribution of detected Gram-negative pathogens in KT recipients

For a detailed overview of the prevalence of the main Gram-negative pathogens, see Table 2. Specifically, *E. coli* was identified in 2141 of 5215 cases (41%). The absolute detection of isolates slightly declined during the years of the COVID-19 pandemic, while the relative proportion remained stable. *Klebsiella spp.* accounted for 613 of 5215 cases (11.7%). Here too, the percentage distribution remained stable during the observation period. *Proteus mirabilis* was isolated in 407 cases (7.8%), *Pseudomonas aeruginosa* was identified in 230 cases (4.3%). A significant difference in the prevalence of the analyzed pathogens was not detected over the study period.

**Table 2:**
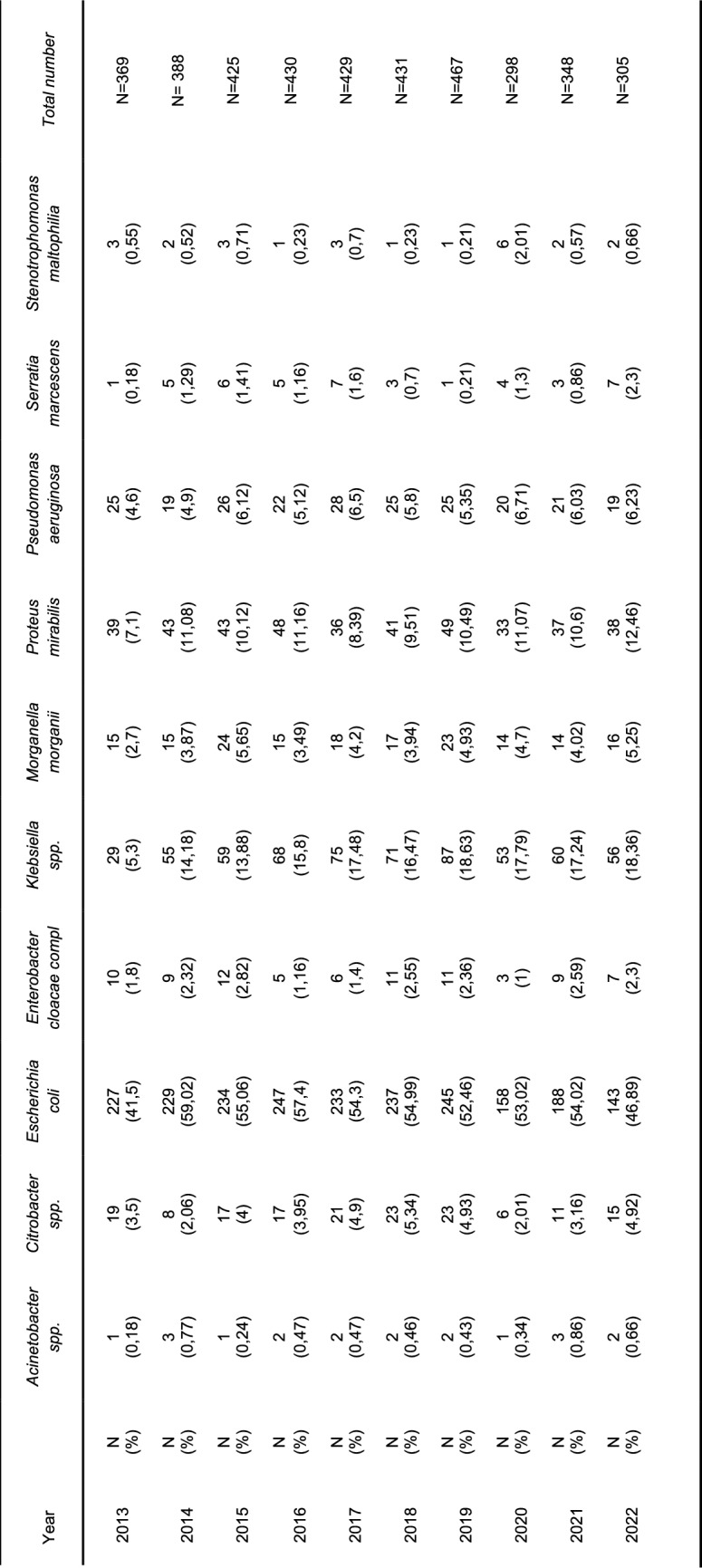
Isolated Gram-negative pathogens 2013–2022

### Distribution of detected Gram-positive pathogens in KT recipients

*Enterococcus spp.* are also common causes of UTI in transplant recipients, accounting for up to 19,4%.

The most frequently isolated Gram-positive pathogen was *E. faecalis* 1012/1325 (76%).

A complete overview of the isolated Gram-positive pathogens is provided in Table 3.

**Table 3:**
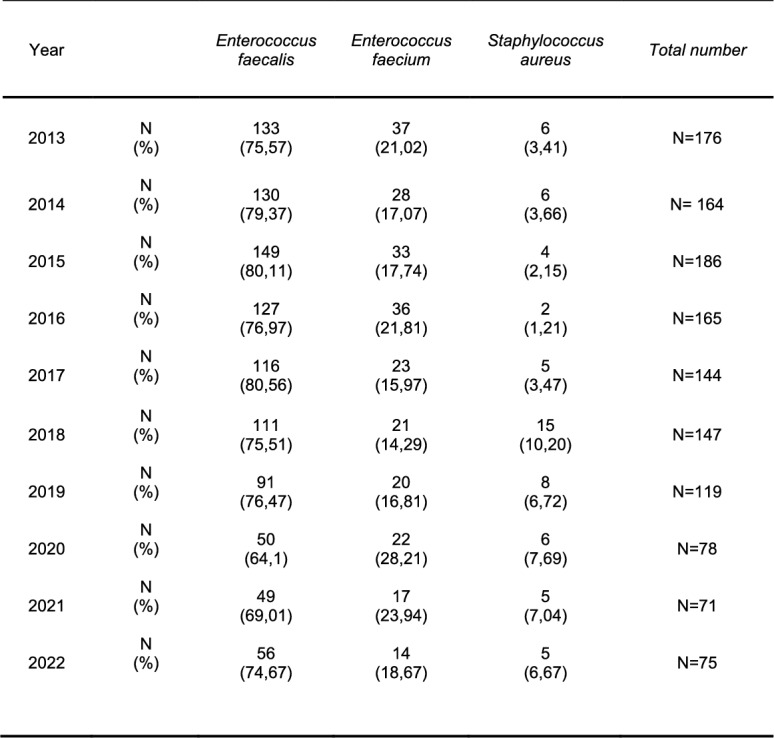
Isolated Gram-positiv pathogens 2013–2022

### Defined daily doses (DDD) of prescribed antibiotics from 2017 to 2023

**Fig. 1:**
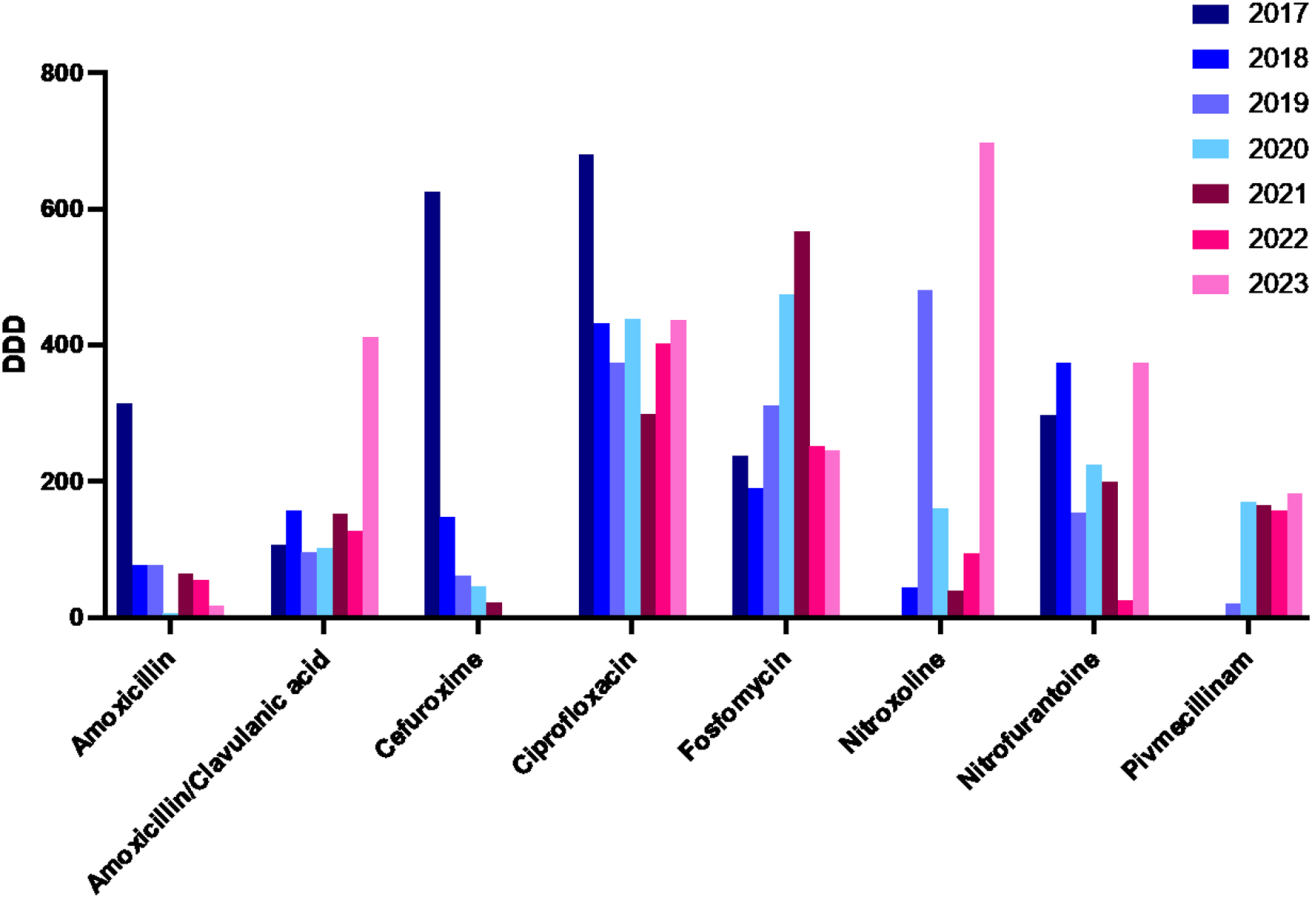
Defined daily doses (DDD) of prescribed antibiotics from 2017 to 2023

During the period from 2017 to 2023, a total of 33,471.49 DDD were prescribed. The most frequently prescribed antibiotic was TMP-SMX, totaling 21,311.58 DDD (63% of total prescriptions). TMP-SMX was almost exclusively prescribed for the prophylaxis of Pneumocystis pneumonia following recent transplantation or after a rejection episode and played no relevant role in the treatment of UTIs. Other commonly prescribed antibiotics included ciprofloxacin and fosfomycin. While oral cephalosporins such as cefuroxime were relatively frequently prescribed in 2017, they have not been used at all since 2022. The importance of substances that have been recommended for the treatment of UTIs in recent years, such as nitroxoline, nitrofurantoin, and pivmecillinam, has increased. The use of amoxicillin/clavulanic acid has increased in 2023. Some agents were prescribed very rarely and are not explicitly listed here. A detailed overview is provided in Supplementary Table 1 and Fig. 1.

### Antimicrobial resistance of *Klebsiella spp., E. coli* and *Pseudomonas aeruginosa*

Antibiotic resistance among Gram-negative uropathogens in KT recipients has generally increased over time in our study, with notable rises in resistance to piperacillin/tazobactam and ceftazidime in *Klebsiella spp.,* and persistent high resistance in *E. coli* to TMP-SMX.

**Fig. 2:**
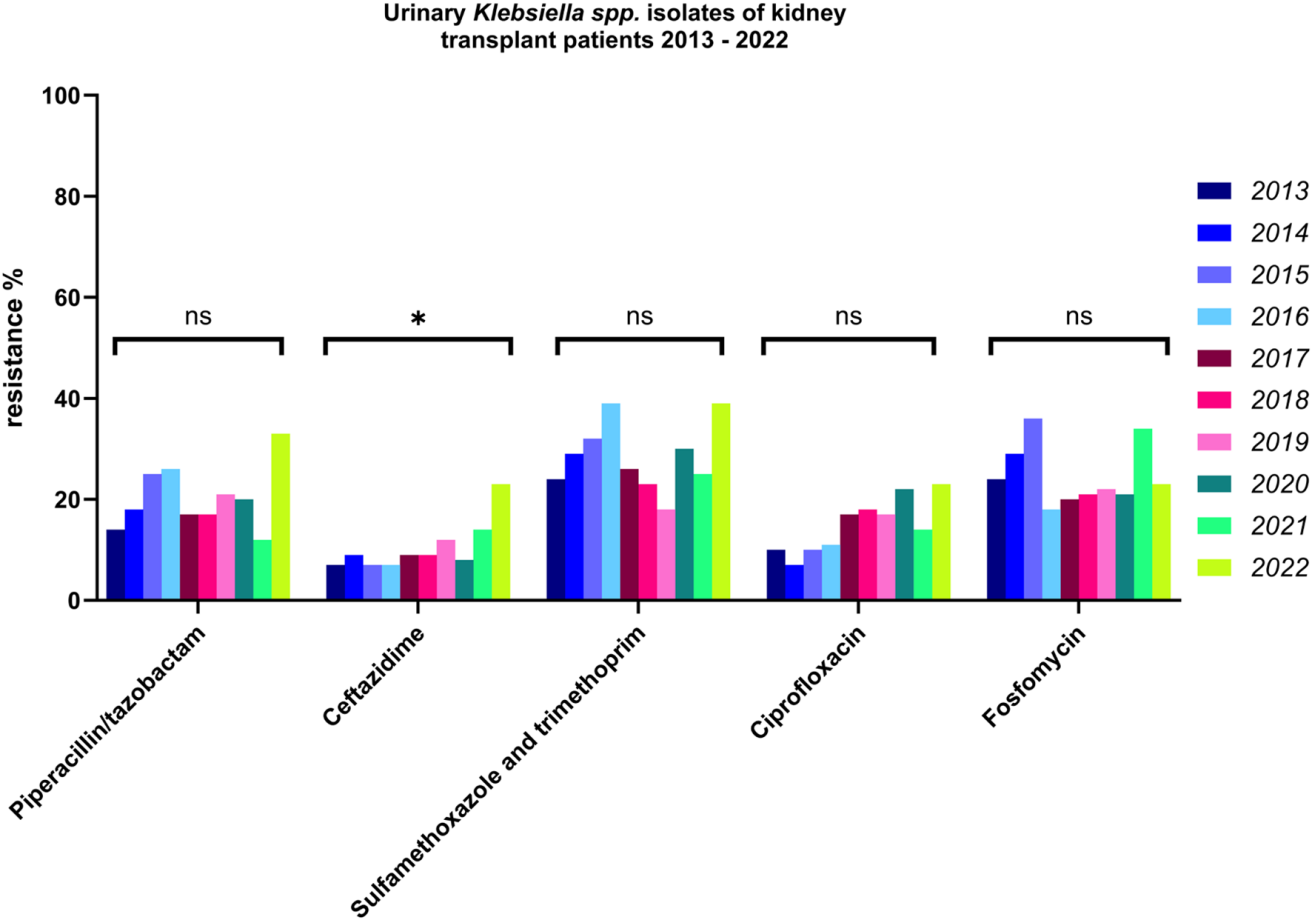
Antibiotic resistance of *Klebsiella* spp. Isolates 2013–2022. Significance: *p < 0.05 (chi-square test)

For *Klebsiella spp.* (Fig. 2) the resistance rate against piperacillin/tazobactam showed a general upward trend, with an increase in 2022 (p = 0.21). The resistance rate against ceftazidime was relatively low, but showed an increase in recent years, particularly in 2022 (p = 0.04). The resistance against TMP-SMX fluctuated over the years, with higher resistance rates in the early and late years of the observed period (p = 0.09). The resistance rate against ciprofloxacin showed an upward trend, especially in 2019 and 2020, and remained relatively high until 2022 (p = 0.4). Resistance to fosfomycin varied overall, but it is generally considered moderate high (p = 0.28). Since there is no EUCAST recommendation for susceptibility testing of pivmecillinam, testing cannot be performed.

**Fig. 3:**
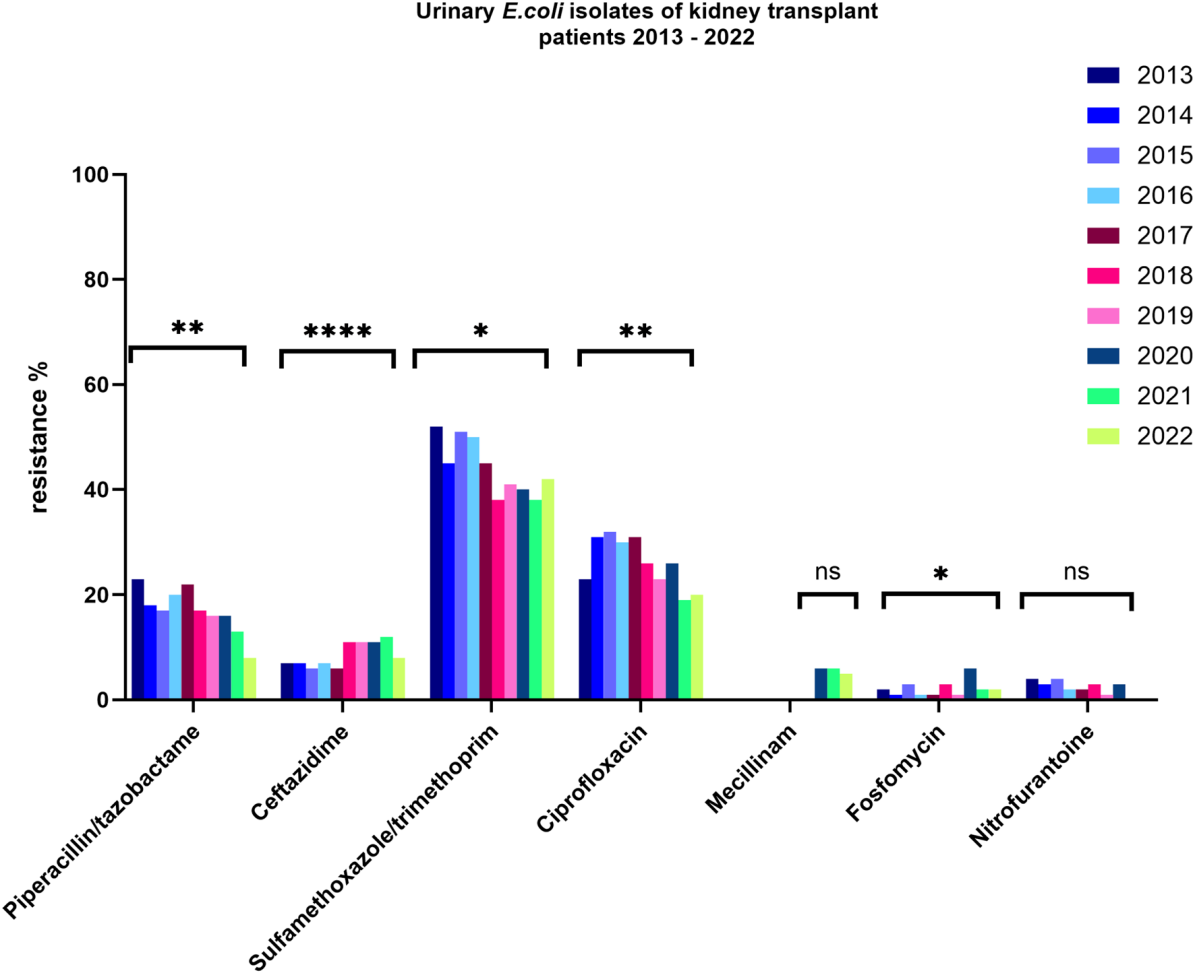
Antibiotic resistance of *E. coli* isolates 2013–2022. Significance: *p < 0.05, **p < 0.01, ***p < 0.001, ****p < 0.0001 (chi-square test)

*E. coli* (Fig. 3) showed moderate resistance rates to piperacillin/tazobactam with a slight decline in recent years (p < 0.01). To ceftazidime low resistance rates were detected that remained fairly constant over the years (p < 0.01). *E. coli* showed high and constant resistance rates against TMP-SMX (p = 0.01). Against ciprofloxacin moderate resistance rates with a slight decline in recent years were shown (p < 0.01). Resistance to fosfomycin were generally low (p = 0.02).

*Pseudomonas aeruginosa* (Supplementary Fig. 1) showed moderate and stable resistance rates to piperacillin/tazobactam (p = 0.23). The resistance to ceftazidime was moderate and remains constant (p = 0.14). To ciprofloxacin *P. aeruginosa* presented fluctuating resistance rates with a general decline (p = 0.39). There is a natural resistance to TMP-SMX and fosfomycin, so an evaluation is not meaningful.

### Antimicrobial susceptibility of *Enterococcus faecalis and Enterococcus faecium*

**Fig. 4:**
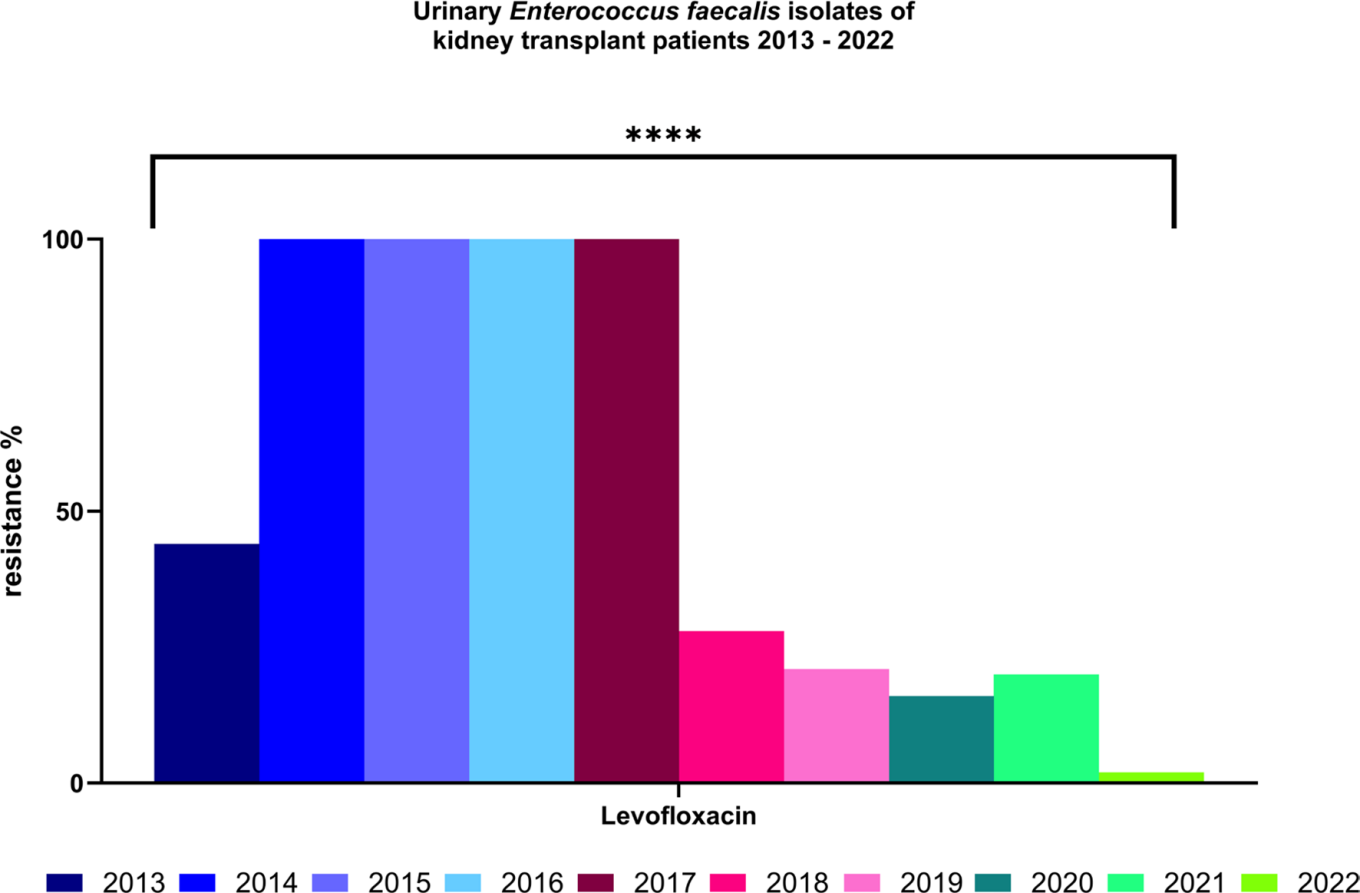
Resistance to levofloxacin of *Enterococcus faecalis* isolates 2013–2022. Significance: *p < 0.05, **p < 0.01, ***p < 0.001, ****p < 0.0001 (chi-square test)

For *Enterococcus faecalis* (Fig. 4) exhibited consistently low resistance rates to ampicillin, vancomycin and linezolid throughout the study period. Interestingly, resistance for levofloxacin, previously high, significantly declined starting in 2018, dropping from 100% in 2014–2017 to just 2% in 2022 (p < 0.01).

*Enterococcus faecium* (Supplementary Fig. 2) showed very low resistance rates to linezolid. However, the resistance to vancomycin increased over time with rates rising from 16% in 2013 to 33% in 2018, before stabilizing at 14% in 2022 (p = 0,28).

### Development of multidrug resistant organisms

**Fig. 5:**
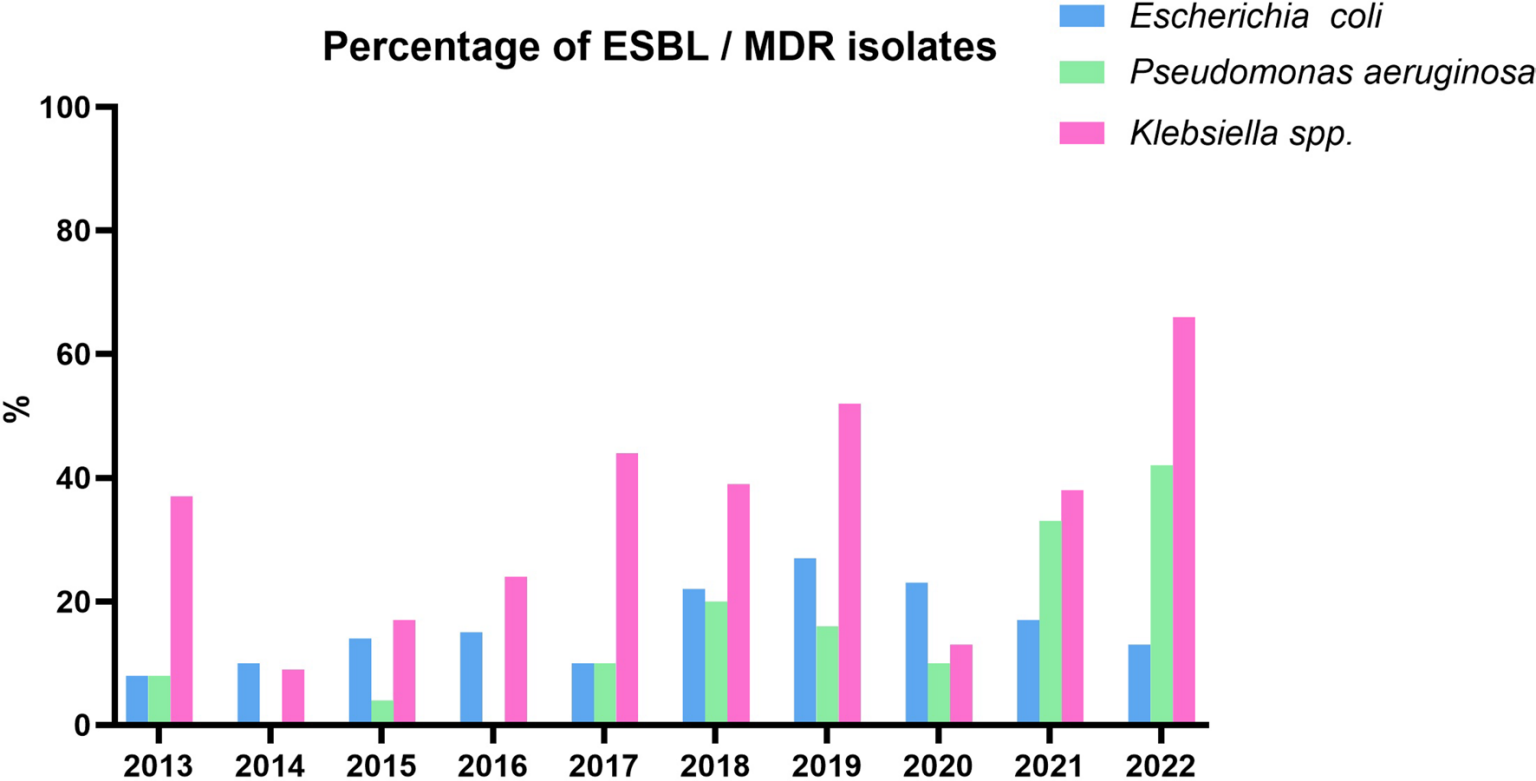
Percentage of ESBL and MDR isolates 2013–2022

Regarding Gram negative organisms, we focused our analysis on ESBL-producing *E. coli* and *Klebsiella spp.*, and MDR *P. aeruginosa*. A detailed overview is provided in Fig. 5, Table 4 and Supplementary Figs. 3–5.

**Table 4:** Multidrug resistant isolates from 2013–2022 absolute

Year		*Escherichia coli *(ESBL)	*Klebsiella spp. *(ESBL)	*Pseudomonas aeruginosa *(MDR)
2013	N (%)	19 (8)	11 (37)	2 (8)
2014	N (%)	24 (10)	5 (9)	0 (0)
2015	N (%)	33 (14)	10 (17)	1 (4)
2016	N (%)	36 (15)	16 (24)	0 (0)
2017	N (%)	23 (10)	33 (44)	3 (10)
2018	N (%)	53 (22)	28 (39)	5 (20)
2019	N (%)	66 (27)	45 (52)	4 (16)
2020	N (%)	37 (23)	7 (13)	5 (10)
2021	N (%)	32 (17)	23 (38)	7 (33)
2022	N (%)	18 (13)	37 (66)	8 (42)

In parentheses: percentage share of all ESBL / MDR isolates within a pathogen group within a year

In *E. coli*, both the absolute numbers and the percentage of ESBL cases increased until 2020, after which a declining trend has been observed. For *Klebsiella spp.*, aside from a slight decrease in 2020, there has generally been an increasing trend in ESBL development.

For *P. aeruginosa*, although the absolute numbers are low, an increase in MDR cases is also evident.

### Urosepsis and transplant pyelonephritis

Of all the recorded cases, based on the ICD codes, 163 cases were identified as hospital admissions with the diagnosis of urosepsis (A41), 368 cases with transplant pyelonephritis (N39) following a positive urinary culture. In sepsis cases, the results of submitted blood and urine cultures were further analyzed. An overview of the detected pathogens in blood or urine cultures is provided in Supplementary Table 2. Bloodstream infections were most frequently caused by Gram-negative pathogens. *E. coli* was the most detected pathogen in both cases. Enterococci were also frequently found, especially in urine cultures.

## Discussion

This study investigated antimicrobial resistance in Gram-negative and Gram-positive uropathogens among KT recipients over one decade (2013–2022). *Enterobacterales*, particularly *E. coli* and *Klebsiella* spp., accounted for 60% of bacteriuria cases, consistent with other cohorts [16]. Resistance rates in Gram-negative pathogens, especially *Klebsiella* spp., have increased significantly.

*Klebsiella spp.* colonizes renal transplant patients more frequently than healthy individuals*.* Our data show a notable increase in resistance of *Klebsiella spp.* to both piperacillin/tazobactam and ceftazidime. This increase is markedly higher compared to national German surveillance data, where resistance rates in the general outpatient population are reported to be up to 13.9% for ceftazidime and 11.9% for piperacillin/tazobactam [17]. The proportion of ESBL-producing isolates has also surged, with fluctuations during the pandemic period. Further monitoring of the cohort in the coming years is necessary and important, given that resistance in *Klebsiella spp*. is already a global concern [18].

*E. coli* often causes UTIs both in KT recipients and non-transplant patients and showed high and stable resistance to TMP-SMX in our cohort. *E. coli* remains the most common Gram-negative pathogen causing UTI, as observed in the studied cohort [19]. Resistance was primarily detected for TMP-SMX and ciprofloxacin, with lesser relevance for piperacillin/tazobactam and ceftazidime. The relatively high resistance rate to TMP-SMX may be related to the local guidelines for the treatment of KT recipients. During the observation period, there was a downward trend in both the DDD of TMP-SMX (−56,58% in 2017 compared to 2013) and the resistance of *E. coli* (52% in 2013 compared to 42% in 2022). Another influencing factor could be the decreasing number of kidney transplants in Germany (2015: 648; 2022: 505; a reduction of 22%) [20]. The decline in ESBL-producing *E. coli* since the COVID-19 pandemic may reflect effective AMS measures.

For *P. aeruginosa*, stable resistance data were observed for piperacillin/tazobactam and ceftazidime. There was a decrease in resistance against ciprofloxacin.

For ESBL and MDR pathogens, *E. coli* showed a decline post-pandemic, while *Klebsiella spp.* and *P. aeruginosa* exhibited rising trends. *Klebsiella spp.* are becoming an increasing problem. This trend is also reflected in other studies from different cohorts [19]. Biofilm formation in *Klebsiella* spp. enhances resistance through limited antibiotic penetration and horizontal gene transfer, increasing clinical challenges. ESBL infections often require carbapenem therapy, associated with higher costs, longer hospital stays, and increased mortality [21].

In a seminal study, Goossens and colleagues demonstrated a significant positive association between the outpatient use of antibiotics and the extent of antimicrobial resistance among *E. coli* infections on a Europe-wide scale [22]. Particularly KT recipients with recurrent UTIs appear to have an increased risk of infections with multidrug-resistant Gram-negative pathogens [23].

*E. faecalis* and *E. faecium* showed a very low resistance against linezolid, indicating its continued effectiveness. While the detection of enterococci in urine is often considered colonization, it is important to note that enterococci can act as true pathogens in immunocompromised populations. In this vulnerable group, enterococcal infections can lead to significant morbidity, especially when associated with complicated UTIs, bacteremia, or graft-related complications [24]. Distinguishing between colonization and true infection represents a major clinical challenge in the management of infections in transplant recipients. This diagnostic uncertainty often complicates treatment decisions, particularly regarding the initiation or discontinuation of antibiotic therapy [25].

A significant decrease in levofloxacin resistance in *E. faecalis* was observed, dropping from 100% in 2014 to 2% in 2022. Although fluoroquinolones are not recommended as first-line therapy for enterococcal infections due to limited clinical efficacy, particularly against *E. faecium*, they were included in our analysis to reflect prescribing trends and to assess the impact of AMS measures over time.

A general trend of decreasing resistance to fluoroquinolones can also be observed in *Pseudomonas aeruginosa*, *E. coli*, and Enterococci. This suggests that adherence to recommended treatment guidelines can be associated to antibiotic prescription practices and the development of resistance. Following a safety warning in 2019 regarding the use of fluoroquinolones, a reduction in resistance was already observed the subsequent year.

Bloodstream infections were rare, primarily caused by Gram-negative pathogens like *E. coli*, with 36 out of 163 cases due to MDR organisms, which is considered relatively high. This can be attributed to several factors specific to the KT population. Chronic immunosuppression increases susceptibility to infections with opportunistic and resistant pathogens. Additionally, KT recipients often experience recurrent UTIs, leading to repeated courses of broad-spectrum antibiotics. This contributes to selective pressure, favoring the emergence and persistence of MDR organisms [26].

When assessing the development of resistance in uropathogenic bacteria over such a long period, it is important to consider how treatment guidelines have evolved in recent years. In the early 2010s the AMS Movement became more and more important [27]. Since 2016, fosfomycin was established as the preferred first-line treatment for uncomplicated UTIs to improve treatment safety and efficacy while reducing resistance. This shift was part of the broader AMS initiative as well and was also reflected in the German recommendations for the treatment of UTIs in adults issued in 2017 [28, 29]. It emphasizes evidence-based treatment and promoting responsible antibiotic use to provide clear recommendations and minimize resistance. At our center’s KT outpatient clinic, there has been an increase in fosfomycin prescriptions since 2017. The resistance situation remains favorable for most Gram-negative pathogens. A recent study from South Africa also reported low fosfomycin resistance rates, aligning with global trends [30]. This serves as an indication of how guidelines can positively influence clinical practice. Since 2018 recommendations were updated to discourage routine treatment of ASB with antibiotics aiming to prevent unnecessary antibiotic use and reduce resistance risk [31].

A very significant event in Germany was the issuance of the *Rote Hand Brief* (Red-Hand Letter) in April 2019 and FDA warning update in 2018. They were issued warning of the serious and potentially irreversible side effects of fluoroquinolones. This led to strict restrictions on their use, recommending them only after careful benefit-risk assessment [32]. In the KT outpatient clinic of our center, the use of fluoroquinolones has tended to decrease. Additionally, the use of non-antibiotic medications such as phytotherapeutics has gained relevance in outpatient care in recent years [33, 34].

The COVID-19 pandemic abruptly changed all our lives, inevitably leading to a massive reduction in travel and enhanced hygiene measures that, for the first time, also strongly impacted the private sector. This influenced the development of resistance, which is also reflected in our cohort [35].

Another factor that has influenced the development of resistance in recent years, especially in Germany and Europe, is the movement of refugees from Syria, Ukraine, Russia, and Afghanistan. For various reasons, there is already a higher prevalence/incidence of multidrug-resistant organisms in these countries of origin, particularly carbapenemase-producing *Enterobacterales* [36].

Overall, it is positive to note that fosfomycin, nitroxoline, nitrofurantoin, and pivmecillinam—which should continue to be used as first-line therapy for uncomplicated UTIs according to the current European recommendations on urological infections—also show a favorable resistance profile against *Enterobacterales* in our cohort [37]. Their use in patients after KT therefore appears to be safe and is already routinely applied. There was a significant increase in the prescription of pivemecillinam since the guideline change in 2017 (2017: 0 DDDs, 2023: 183.334; p < 0.01). The use of pivmecillinam for UTIs is still recommended due to the continuously favorable resistance situation [38, 39]. This underscore again how guideline recommendations can positively influence clinical practice and highlights the importance of continuously conducting new surveys on resistance development to keep the recommendations up to date.

An exception here is *Klebsiella spp.*, which already shows an increasing development of resistance to the oral first-line substances. It remains to be seen whether new substances such as gepotidacin, which have shown good efficacy in current studies on uncomplicated UTIs, can also be safely used in the KT population [40].

The prescription of nitroxoline and nitrofurantoin in our study was very variable. These substances are primarily used for the prophylaxis of recurrent UTIs. Some outliers, such as the one for nitroxoline in 2022, are due to prescriptions for individual patients who received such prophylaxis. Additionally, nitroxoline was unavailable from March to July 2021 due to supply shortages [41].

Moreover, it is positive that the use of oral cephalosporins at our outpatient clinic has significantly declined. It is now known that oral administration results in insufficient therapeutic levels [42]. Oral cephalosporins promote the development of *Clostridoides difficile* infections and MRSA colonization [43].

However, it’s important to note that resistance patterns vary significantly depending on the region.

A large surveillance study from Germany investigated the resistance of *Enterobacterales* in urine samples between 2016 and 2021. A subgroup analysis was conducted among young women, postmenopausal women, and men [44]. Overall, a decrease in resistance was observed regardless of age and sex. However, the resistance data differ from our cohort, highlighting the need for specialized treatment considerations for KT recipients due to their unique risk factors.

High resistance rates in Gram-negative bacteria have been reported globally, with studies from the Asia–Pacific and the Middle East showing MDR prevalence rates of up to 80%, underscoring the importance of national surveillance programs to protect public health and guide treatment strategies [45, 46]. The major challenge of antibiotic resistance can only be resolved within the international community.

Our study has several limitations. Although the observation period is long and the number of samples analyzed is high at 10,508, only outpatients from a single KT outpatient clinic were included. Furthermore, treatment strategies changed during the observation period, and we only had antibiotic consumption data from 2017 onwards. While a direct, individual-level linkage between microbiological data and antibiotic prescriptions was not feasible due to system constraints, targeted data assignment based on health insurance records enabled a robust correlation between antibiotic use and resistance trends across the entire cohort of KT outpatients. Therefore, more frequent monitoring of resistance development for the KT patient collective would be beneficial in the future.

Since we conducted a case-based rather than a patient-based evaluation, individual patients may have been included multiple times in the analysis. The evaluation regarding hospitalization, complicated UTIs, and sepsis was carried out exclusively based on ICD codes for patients treated as inpatients at our center. Patients who were not treated at our center during the observation period or were coded under a different primary diagnosis may therefore not have been captured.

An interesting aspect to consider in future studies is the role of new diagnostic technologies and molecular methods in monitoring antibiotic resistance. While traditional culture methods remain the gold standard, molecular techniques such as PCR (Polymerase Chain Reaction) and Next-Generation Sequencing (NGS) enable faster and more accurate identification of resistant genes and pathogens. There are already reviews for UTIs that attest to early diagnostic accuracy [47]. Implementing these technologies in clinical practice may present challenges, including the need for additional training for medical personnel and the costs associated with acquiring and operating the equipment. However, the long-term benefits, such as improved patient outcomes and reduced treatment costs through more targeted therapy approaches, could justify these initial investments.

## Conclusion

Our study highlights the significant burden of antimicrobial resistance in KT recipients, with notably high rates of MDR organisms, particularly among *Klebsiella spp*. The observed increase in resistance to key antibiotics, such as piperacillin/tazobactam and ceftazidime in *Klebsiella spp.*, underscores the urgent need for targeted AMS interventions in this vulnerable population. The sharp decline in levofloxacin resistance among *E. faecalis* from 100% in 2014 to 2% in 2022 illustrates the positive impact of recommendation adherence and AMS programs on resistance trends. Continuous surveillance is essential to detect emerging resistance patterns early and to guide evidence-based adjustments in clinical practice.

Given the unique susceptibility of KT recipients to severe infections and the potential for graft-related complications, our findings support the need for tailored infection management strategies. These results can inform future guidelines and policies by emphasizing the importance of implementing risk-based AMS specific to transplant populations, regularly updating treatment recommendations based on local resistance data, and promoting strategies to minimize unnecessary antibiotic use, particularly in asymptomatic bacteriuria.

By integrating these insights, future guidelines can help preserve the efficacy of existing antibiotics, optimize patient outcomes, and reduce the burden of MDR infections in transplant recipients.

## Supplementary Information

Below is the link to the electronic supplementary material.

Supplementary file1 (DOCX 19 KB)

Supplementary file2 (DOCX 1566 KB)

## Data Availability

No datasets were generated or analysed during the current study.

## References

[1] Adamska Z, et al. Bacterial infections in renal transplant recipients. Transpl Proc. 2015;47(6):1808–12. 10.1016/j.transproceed.2015.03.046. 10.1016/j.transproceed.2015.03.046 26293055

[2] Witzke O. Impact of febrile infections on the long-term function of kidney allografts. J Urol. 2001;166(6):2048–52. 11696704

[3] Alotaibi NE. Incidence and risk factors of infections following kidney transplantation. J Infect Public Health. 2024;17(8):102491. 10.1016/j.jiph.2024.102491. (**Epub 2024 Jul 2**). 38996795 10.1016/j.jiph.2024.102491

[4] Brune JE, Swiss Transplant Cohort Study, et al. Frequency and impact on renal transplant outcomes of urinary tract infections due to extended-spectrum beta-lactamase-producing *Escherichia coli* and *Klebsiella *species. Front Med (Lausanne). 2024;11:1329778. 10.3389/fmed.2024.1329778. 38426162 10.3389/fmed.2024.1329778 PMC10902035

[5] Hamilton AD, et al. Reduced graft survival in renal transplant patients with urinary tract infections—a meta-analysis. Dan Med J. 2024;71(2):A06230424. 10.61409/A06230424. 38314732 10.61409/A06230424

[6] Veroux M. Infective complications in renal allograft recipients: epidemiology and outcome. Transpl Proc. 2008;40(6):1873–6. 10.1016/j.transproceed.2008.05.065. 10.1016/j.transproceed.2008.05.065 18675076

[7] Al Midani A. Impact of urinary tract infections in kidney transplant recipients: a 4-year single-center experience. Transpl Proc. 2018;50(10):3351–5. 10.1016/j.transproceed.2018.08.022. (**Epub 2018 Sep 7**). 10.1016/j.transproceed.2018.08.022 30577206

[8] Vidal E, et al. Spanish network for research in infectious diseases (REIPI). Bacterial urinary tract infection after solid organ transplantation in the RESITRA cohort. Transpl Infect Dis. 2012;14(6):595–603. 10.1111/j.1399-3062.2012.00744.x. (**Epub 2012 Jun 1**). 22650416 10.1111/j.1399-3062.2012.00744.x

[9] Becker S, et al. Harnwegsinfektionen nach Nierentransplantation : Essener Algorithmus zur kalkulierten Antibiotikatherapie [urinary tract infections after kidney transplantation: Essen algorithm for calculated antibiotic treatment]. Urologe A. 2011;50(1):53–6. 10.1007/s00120-010-2470-x. 21174190 10.1007/s00120-010-2470-x

[10] Korth J, et al. Increased resistance of Gram-negative urinary pathogens after kidney transplantation. BMC Nephrol. 2017;18(1):164. 10.1186/s12882-017-0580-z. 28525997 10.1186/s12882-017-0580-z PMC5437586

[11] Strohaeker J, et al. Urinary tract infections in kidney transplant recipients—is there a need for antibiotic stewardship? J Clin Med. 2022;11:226. 10.3390/jcm11010226. 10.3390/jcm11010226 PMC8745876 35011966

[12] Goldman JD, et al. Urinary tract infections in solid organ transplant recipients: guidelines from the American society of transplantation infectious diseases community of practice. Clin Transpl. 2019;33(9):e13507. 10.1111/ctr.13507. (**Epub 2019 Mar 28**). 10.1111/ctr.13507 30793386

[13] Magiorakos A-P, et al. Multidrug-resistant, extensively drug-resistant and pandrug-resistant bacteria: an international expert proposal for interim standard definitions for acquired resistance. Clin Microbiol Infect. 2012;18(3):268–81. 21793988 10.1111/j.1469-0691.2011.03570.x

[14] Tamma PD. Infectious diseases society of America 2022 guidance on the treatment of extended-spectrum β-lactamase producing Enterobacterales (ESBL-E), carbapenem-resistant Enterobacterales (CRE), and *Pseudomonas aeruginosa* with difficult-to-treat resistance (DTR-*P. aeruginosa*). Clin Infect Dis. 2022;75(2):187–212. 10.1093/cid/ciac268. 35439291 10.1093/cid/ciac268 PMC9890506

[15] Fiorentino M, et al. Updates on urinary tract infections in kidney transplantation. J Nephrol. 2019;32:751–61. 10.1007/s40620-019-00585-3. 30689126 10.1007/s40620-019-00585-3

[16] Krawczyk B, et al. Urinary tract infections caused by *K. pneumoniae* in kidney transplant recipients—epidemiology, virulence and antibiotic resistance. Front Cell Infect Microbiol. 2022;12:861374. 10.3389/fcimb.2022.861374. 35531341 10.3389/fcimb.2022.861374 PMC9068989

[17] https://amr.rki.de/Content/Datenbank/ARS/ResistanceOverview.aspx. Accessed 10 May 2024.

[18] Zhang H, et al. Antimicrobial resistance comparison of *Klebsiella pneumoniae* pathogens isolated from intra-abdominal and urinary tract infections in different organs, hospital departments and regions of China between 2014 and 2017. J Microbiol Immunol Infect. 2021;54(4):639–48. 10.1016/j.jmii.2020.03.009. 32247662 10.1016/j.jmii.2020.03.009

[19] Thomas A. Urinary tract infection antibiotic resistance in the United States. Prim Care Clin Off Pract. 2018;45(3):455–66. 10.1016/j.pop.2018.05.005 30115334

[20] https://www.dso.de/organspende/statistiken-berichte/organspende. Accessed 15 May 2024.

[21] https://www.frontiersin.org/journals/medicine/articles/, 10.3389/fmed.2024.1329778/full. Accessed 19 May 2024.

[22] Goossens H, ESAC Project Group, et al. Outpatient antibiotic use in Europe and association with resistance: a cross-national database study. Lancet. 2005;365(9459):579–87. 10.1016/S0140-6736(05)17907-0. 15708101 10.1016/S0140-6736(05)17907-0

[23] Gangula RS, et al. Effect of urinary tract infection on the outcome of the allograft in patients with kidney transplantation. J Bras Nefrol. 2024;46(4):e20240002. 10.1590/2175-8239-JBN-2024-0002en. (**PMID: 39311799**). 39311799 10.1590/2175-8239-JBN-2024-0002en PMC11420934

[24] Gołębiewska JE, Dębska-Ślizień A, Rutkowski B. Urinary tract infections during the first year after renal transplantation: one center’s experience and a review of the literature. Clin Transpl. 2014;28(11):1263–70. 10.1111/ctr.12465. (**Epub 2014 Oct 15 PMID: 25251447**). 10.1111/ctr.12465 25251447

[25] Hollyer I, Ison MG. The challenge of urinary tract infections in renal transplant recipients. Transpl Infect Dis. 2018;20(2):e12828. 10.1111/tid.12828. (**Epub 2018 Jan 25. PMID: 29272071**). 29272071 10.1111/tid.12828

[26] Espinar MJ. Urinary tract infections in kidney transplant patients due to *Escherichia coli* and *Klebsiella pneumoniae*-producing extended-spectrum β-lactamases: risk factors and molecular epidemiology. PLoS ONE. 2015;10(8):e0134737. 10.1371/journal.pone.0134737. 26237422 10.1371/journal.pone.0134737 PMC4523193

[27] O’Brien M. Unmet needs in uncomplicated urinary tract infection in the United States and Germany: a physician survey. BMC Infect Dis. 2023;23:281. 10.1186/s12879-023-08207-x. 37138215 10.1186/s12879-023-08207-x PMC10158246

[28] Gupta K, Hooton TM, Naber KG, Wullt B, Colgan R, Miller LG, Morane GJ, et al. International clinical practice guidelines for the treatment of acute uncomplicated cystitis and pyelonephritis in women: a 2010 update by the infectious diseases society of America and the European society for microbiology and infectious diseases. Clin Infect Dis. 2011;52(5):e103–20. 10.1093/cid/ciq257. 21292654 10.1093/cid/ciq257

[29] https://register.awmf.org/assets/guidelines/043-044k_S3_Harnwegsinfektionen_2017-05.pdf. Accessed 29 May 2024.

[30] Mosime LB. Fosfomycin resistance in community-acquired urinary pathogens from Western Cape, South Africa. S Afr J Infect Dis. 2022;37(1):321. 10.4102/sajid.v37i1.321. 35169586 10.4102/sajid.v37i1.321 PMC8831924

[31] Nicolle LE, et al. Clinical practice guideline for the management of asymptomatic bacteriuria: 2019 update by the infectious diseases society of America. Clin Infect Dis. 2019;68(10):e83–110. 10.1093/cid/ciy1121. 30895288 10.1093/cid/ciy1121

[32] Bundesinstitut für Arzneimittel und Medizinprodukte. Rote-Hand-Brief zu Fluorchinolon-Antibiotika: Schwerwiegende und anhaltende, die Lebensqualität beeinträchtigende und möglicherweise irreversibleNebenwirkungen. 2019. https://www.bfarm.de/SharedDocs/Risikoinformationen/Pharmakovigilanz/DE/RHB/2019/rhb-fluorchinolone.html. Accessed 18 May 2024.

[33] Höller M, et al. Treatment of urinary tract infections with Canephron® in Germany: a retrospective database analysis. Antibiotics. 2021;10:685. 10.3390/antibiotics1006068. 34201264 10.3390/antibiotics10060685 PMC8226679

[34] Jung N, et al. Choosing wisely internationally—helpful recommendations for antimicrobial stewardship! Infection. 2023;51(3):567–81. 10.1007/s15010-023-02005-y. (**Epub 2023 Feb 25**). 36840828 10.1007/s15010-023-02005-y PMC10205825

[35] Langford BJ. Antibiotic resistance associated with the COVID-19 pandemic: a systematic review and meta-analysis. Clin Microbiol Infect. 2023;29(3):302–9. 10.1016/j.cmi.2022.12.006. (**Epub 2022 Dec 9**). 36509377 10.1016/j.cmi.2022.12.006 PMC9733301

[36] Pallett SJC, et al. The contribution of human conflict to the development of antimicrobial resistance. Commun Med (Lond). 2023;3(1):153. 10.1038/s43856-023-00386-7. 37880348 10.1038/s43856-023-00386-7 PMC10600243

[37] Franz J, et al. European association of urology guidelines on urological infections: summary of the 2024 guidelines. Eur Urol. 2024;86(1):27–41. 10.1016/j.eururo.2024.03.035. (**Epub 2024 May 6**). 38714379 10.1016/j.eururo.2024.03.035

[38] Bader MS, et al. Treatment of urinary tract infections in the era of antimicrobial resistance and new antimicrobial agents. Postgrad Med. 2020;132(3):234–50. 10.1080/00325481.2019.1680052. (**Epub 2019 Oct 24**). 31608743 10.1080/00325481.2019.1680052

[39] Frimodt-Møller N, et al. Treating urinary tract infections in the era of antibiotic resistance. Expert Rev Anti Infect Ther. 2023;21(12):1301–8. 10.1080/14787210.2023.2279104. (**Epub 2023 Nov 24**). 37922147 10.1080/14787210.2023.2279104

[40] Watkins RR, et al. Gepotidacin: a novel, oral, ‘first-in-class’ triazaacenaphthylene antibiotic for the treatment of uncomplicated urinary tract infections and urogenital gonorrhoea. J Antimicrob Chemother. 2023;78(5):1137–42. 10.1093/jac/dkad060. 36883591 10.1093/jac/dkad060

[41] Gelbe Liste. Lieferengpass Nitroxolib forte. https://www.gelbe-liste.de/lieferengpaesse/lieferengpass-nitroxolin-forte. Accessed 10 May 2024

[42] Klepser ME, et al. Clinical pharmacokinetics of newer cephalosporins. Clin Pharmacokinet. 1995;28(5):361–84. 10.2165/00003088-199528050-00003. (**PMID: 7614776**). 7614776 10.2165/00003088-199528050-00003

[43] Rafey A. Antibiotics associated with Clostridium difficile infection. Cureus. 2023;15(5):e39029. 10.7759/cureus.39029. 37323360 10.7759/cureus.39029 PMC10266117

[44] Stoltidis-C Aydın S, et al. Five compelling UTI questions after kidney transplant. World J Urol. 2020;38(11):2733–42. 10.1007/s00345-020-03173-4. (**Epub 2020 Apr 7**). 32266510 10.1007/s00345-020-03173-4

[45] Sugianli AK, et al. Antimicrobial resistance among uropathogens in the Asia-Pacific region: a systematic review. JAC Antimicrob Resist. 2021;3(1):dlab003. 10.1093/jacamr/dlab003. 34223081 10.1093/jacamr/dlab003 PMC8210283

[46] Alotaibi BS, et al. Resistance pattern in mostly Gram-negative bacteria causing urinary tract infections. Infect Disord Drug Targets. 2023;23(2): e280922209238. 10.2174/1871526522666220928115043. 36173061 10.2174/1871526522666220928115043

[47] Zhao M, et al. Comparison of polymerase chain reaction and next-generation sequencing with conventional urine culture for the diagnosis of urinary tract infections: a meta-analysis. Open Med (Wars). 2024;19(1):20240921. 10.1515/med-2024-0921. 38584848 10.1515/med-2024-0921 PMC10996999

