# Supplementary material for: Antibiotic resistance of urinary pathogens after kidney transplantation: a 10-year single-center survey in Germany
Source: Infection. 2025 Mar 10;53(5):1755–68. doi: 10.1007/s15010-025-02493-0 (PMC12460382; doi:10.1007/s15010-025-02493-0)
Supplement: Supplementary file 1 — Supplementary file1 (DOCX 19 KB) [file 15010_2025_2493_MOESM1_ESM.docx]

| Year |  | *Acinetobacter spp.* | *Citrobacter spp.* | *Escherichiacoli* | *Enterobacter cloacae compl* | *Klebsiella spp.* | *Morganella morganii* | *Proteus mirabilis* | *Pseudomonas aeruginosa* | *Serratia marcescens* | *Stenotrophomonas maltophilia* | *Total number* |
| --- | --- | --- | --- | --- | --- | --- | --- | --- | --- | --- | --- | --- |
| 2013 | N  (%) | 1  (0,18) | 19  (3,5) | 227  (41,5) | 10  (1,8) | 29  (5,3) | 15  (2,7) | 39  (7,1) | 25  (4,6) | 1  (0,18) | 3  (0,55) | N=369 |
| 2014 | N  (%) | 3  (0,77) | 8  (2,06) | 229  (59,02) | 9  (2,32) | 55  (14,18) | 15  (3,87) | 43  (11,08) | 19  (4,9) | 5  (1,29) | 2  (0,52) | N= 388 |
| 2015 | N  (%) | 1  (0,24) | 17  (4) | 234  (55,06) | 12  (2,82) | 59  (13,88) | 24  (5,65) | 43  (10,12) | 26  (6,12) | 6  (1,41) | 3  (0,71) | N=425 |
| 2016 | N  (%) | 2  (0,47) | 17  (3,95) | 247  (57,4) | 5  (1,16) | 68  (15,8) | 15  (3,49) | 48  (11,16) | 22  (5,12) | 5  (1,16) | 1  (0,23) | N=430 |
| 2017 | N  (%) | 2  (0,47) | 21  (4,9) | 233  (54,3) | 6  (1,4) | 75  (17,48) | 18  (4,2) | 36  (8,39) | 28  (6,5) | 7  (1,6) | 3  (0,7) | N=429 |
| 2018 | N  (%) | 2  (0,46) | 23  (5,34) | 237  (54,99) | 11  (2,55) | 71  (16,47) | 17  (3,94) | 41  (9,51) | 25  (5,8) | 3  (0,7) | 1  (0,23) | N=431 |
| 2019 | N  (%) | 2  (0,43) | 23  (4,93) | 245  (52,46) | 11  (2,36) | 87  (18,63) | 23  (4,93) | 49  (10,49) | 25  (5,35) | 1  (0,21) | 1  (0,21) | N=467 |
| 2020 | N  (%) | 1  (0,34) | 6  (2,01) | 158  (53,02) | 3  (1) | 53  (17,79) | 14  (4,7) | 33  (11,07) | 20  (6,71) | 4  (1,3) | 6  (2,01) | N=298 |
| 2021 | N  (%) | 3  (0,86) | 11  (3,16) | 188  (54,02) | 9  (2,59) | 60  (17,24) | 14  (4,02) | 37  (10,6) | 21  (6,03) | 3  (0,86) | 2  (0,57) | N=348 |
| 2022 | N  (%) | 2  (0,66) | 15  (4,92) | 143  (46,89) | 7  (2,3) | 56  (18,36) | 16  (5,25) | 38  (12,46) | 19  (6,23) | 7  (2,3) | 2  (0,66) | N=305 |
|  |  |  |  |  |  |  |  |  |  |  |  |  |
