# Supplementary material for: Antibiotic resistance of urinary pathogens after kidney transplantation: a 10-year single-center survey in Germany
Source: Infection. 2025 Mar 10;53(5):1755–68. doi: 10.1007/s15010-025-02493-0 (PMC12460382; doi:10.1007/s15010-025-02493-0)
Supplement: Supplementary file 2 — Supplementary file2 (DOCX 1566 KB) [file 15010_2025_2493_MOESM2_ESM.docx]

**Supplementary Information**


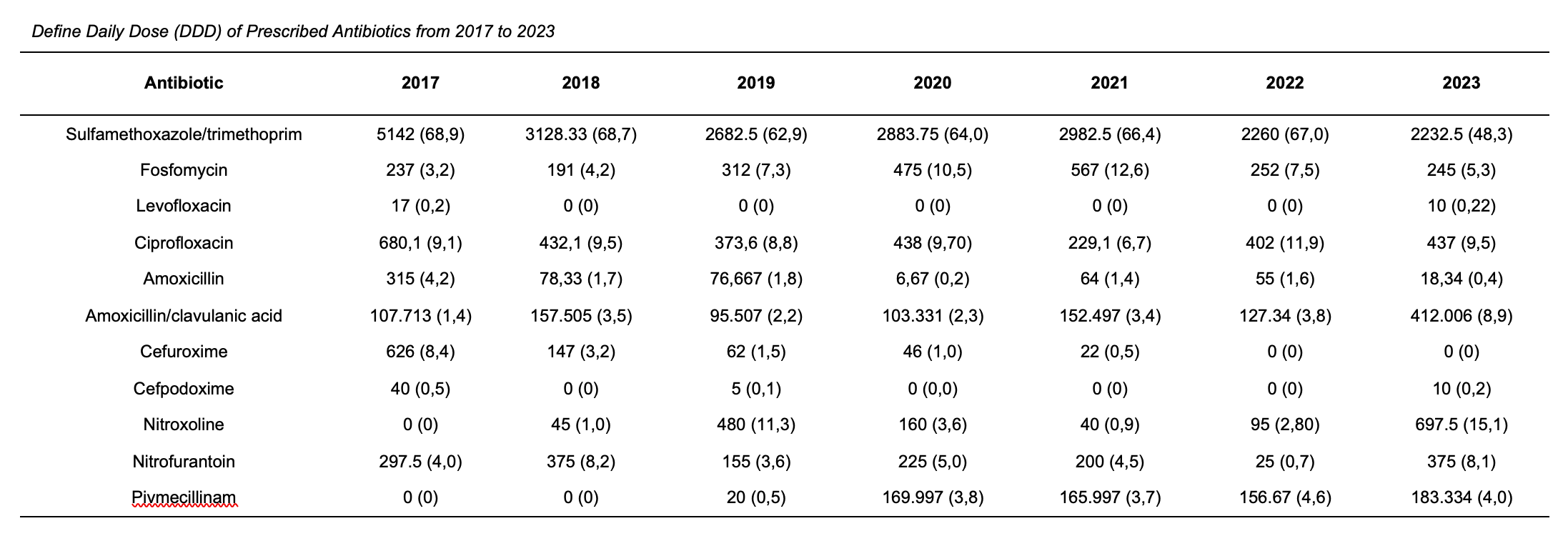


**Supplementary Table 1** Defined Daily Doses (DDD) of Prescribed Antibiotics from 2017 to 2023

In parentheses: percentage share of the DDD within a year


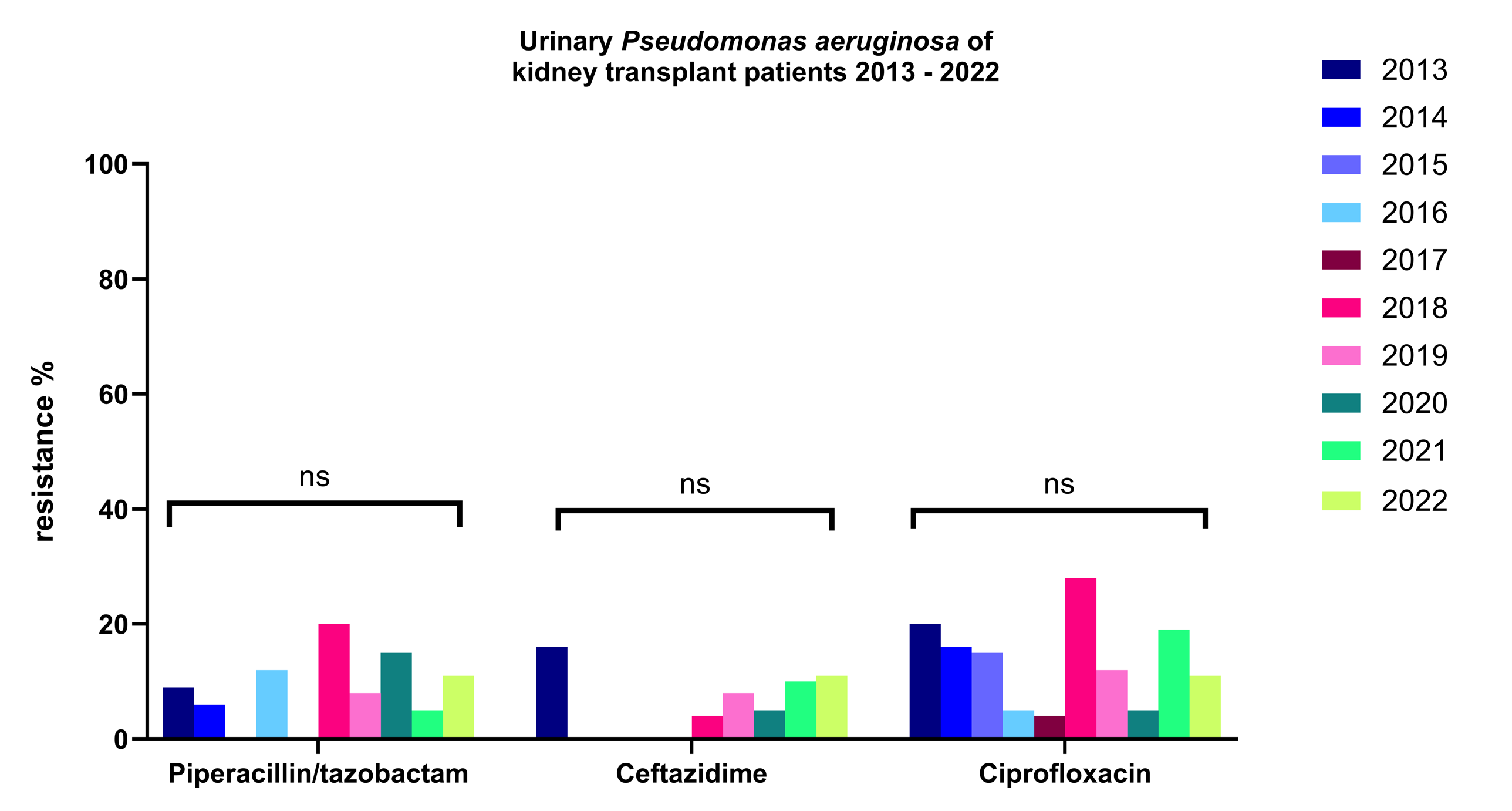


**Supplementary Figure 1** Antibiotic Resistance of Pseudomonas aeruginosa 2013-2022


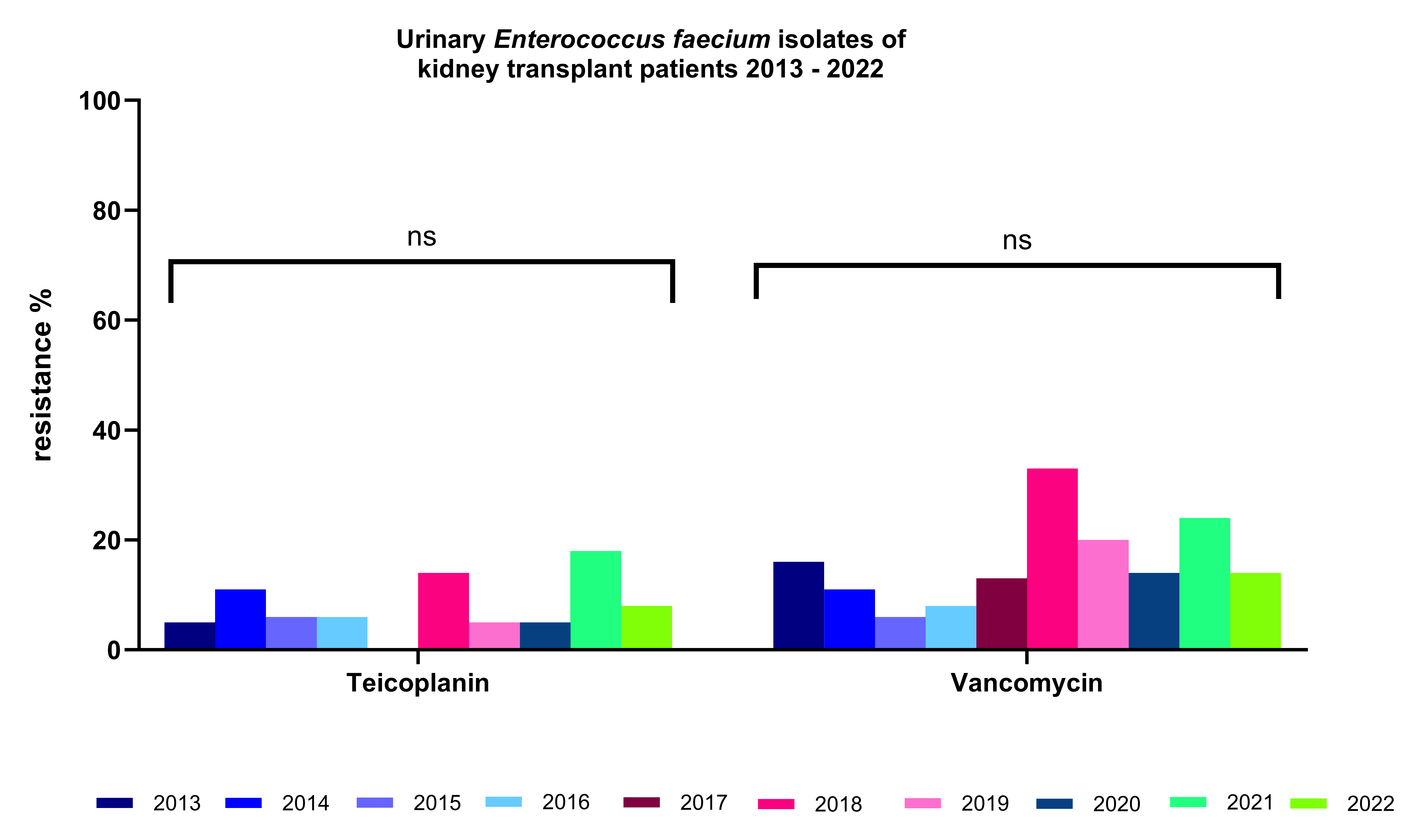


**Supplementary Figure 2** Antibiotic Resistance of Enterococcus faecium 2013-2022


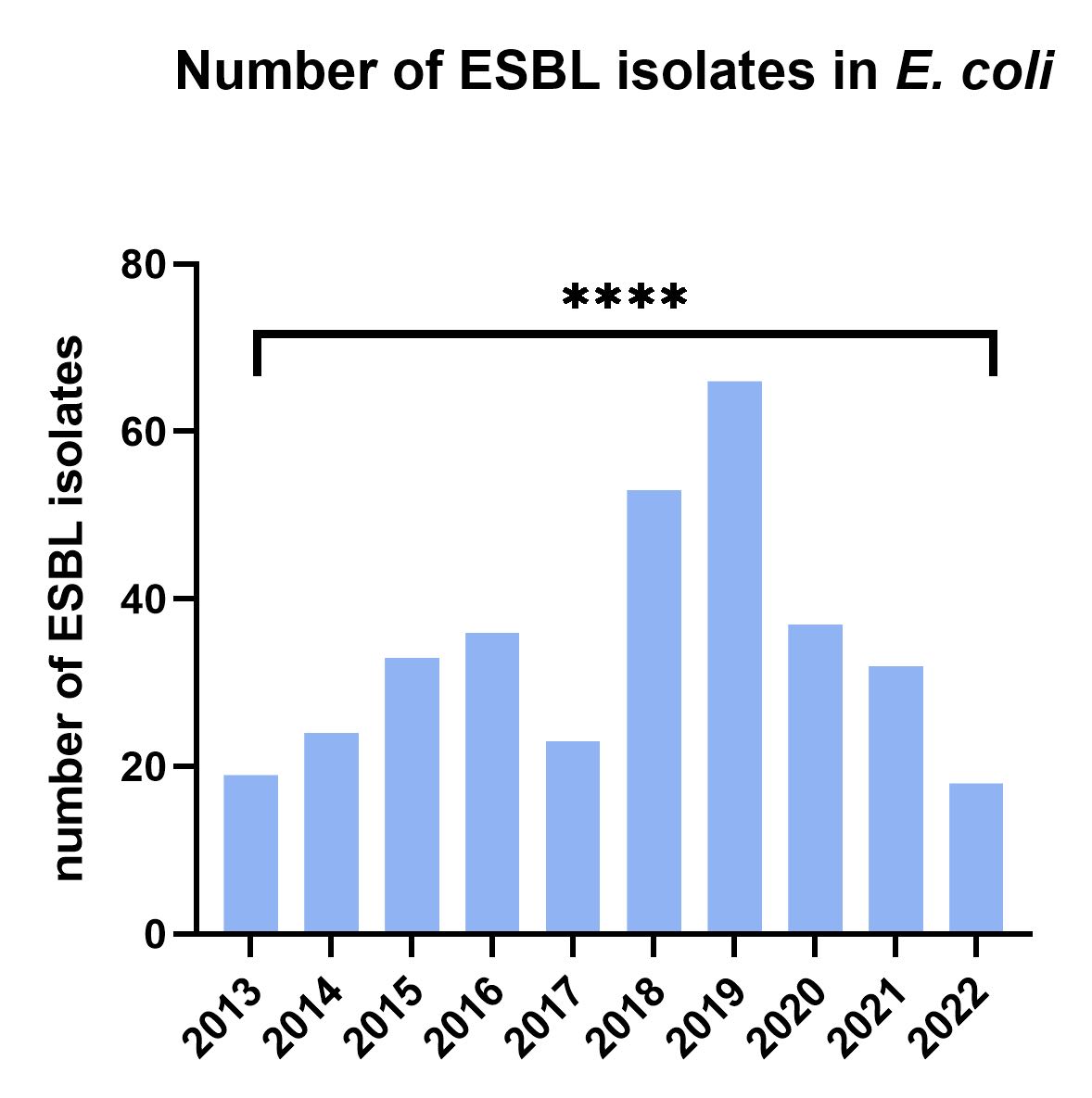


**Supplementary Figure 3** ESBL isolates in E. coli 2013-2022

Significance: **** p < 0.0001 (chi-square test)


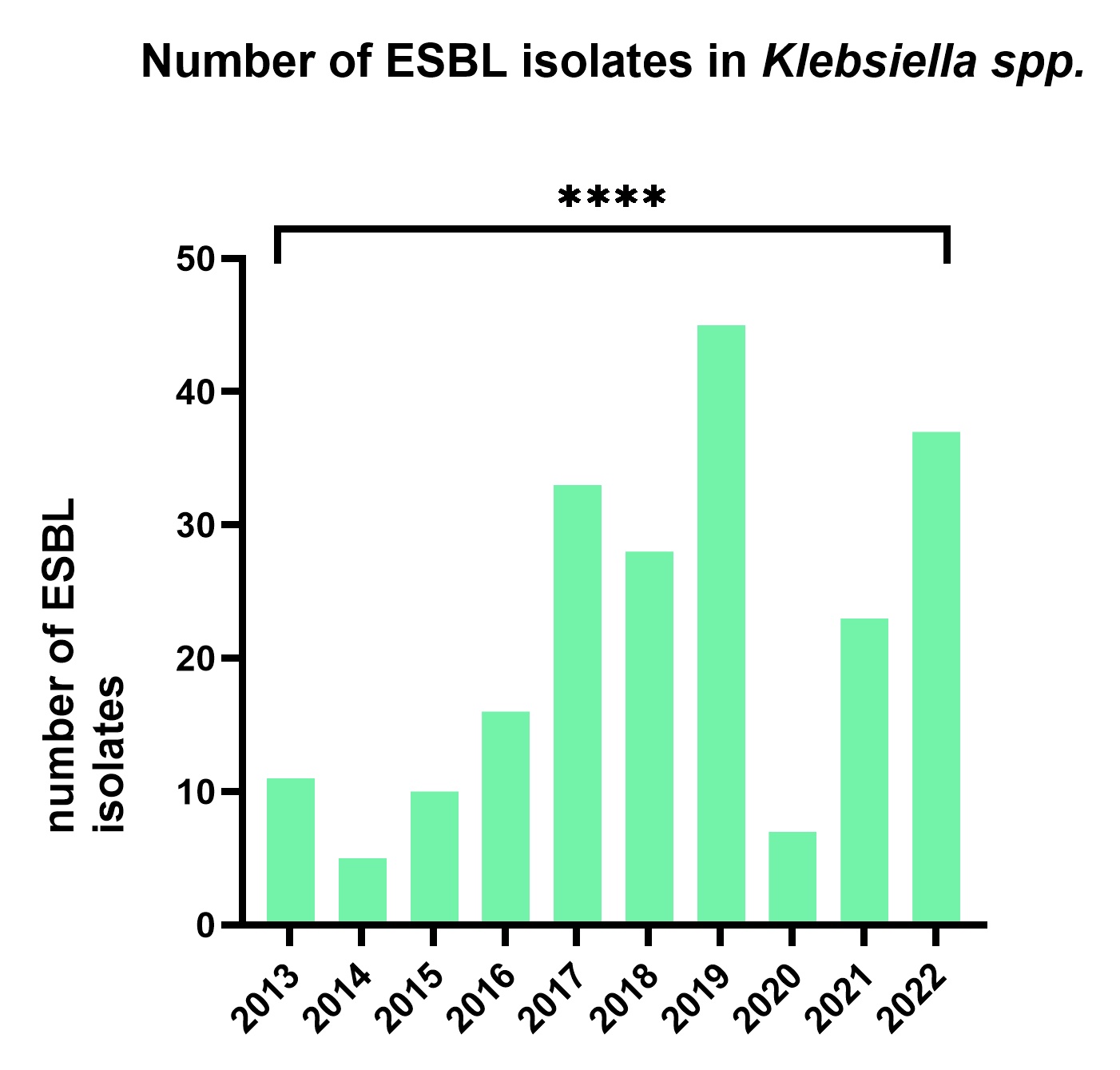


**Supplementary Figure 4** ESBL isolates in Klebsiella ssp. 2013-2022

Significance: **** p < 0.0001 (chi-square test)


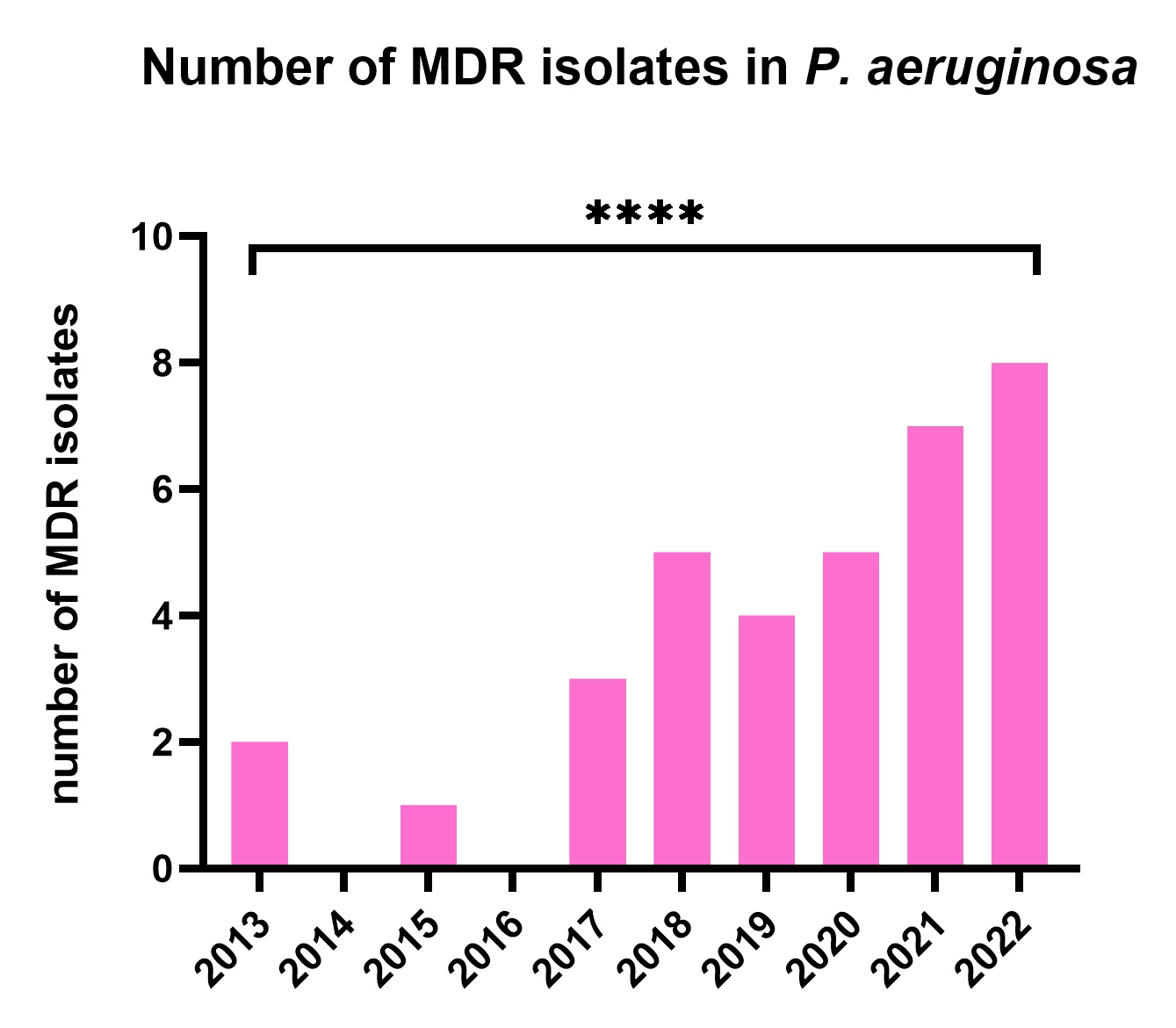


**Supplementary Figure 5** MDR isolates in P. aeruginosa 2013-2022

Significance: **** p < 0.0001 (chi-square test)

**Supplementary Table 2** Sepsis or pyelonephritis pathogens 2013-2022
